# Preferable effects of pemafibrate on liver function and fibrosis in subjects with type 2 diabetes complicated with liver damage

**DOI:** 10.1186/s13098-023-01187-7

**Published:** 2023-10-26

**Authors:** Hiroshi Nomoto, Kenichi Kito, Hiroshi Iesaka, Takahisa Handa, Shingo Yanagiya, Aika Miya, Hiraku Kameda, Kyu Yong Cho, Jun Takeuchi, So Nagai, Ichiro Sakuma, Akinobu Nakamura, Tatsuya Atsumi

**Affiliations:** 1 Department of Rheumatology, Endocrinology and Nephrology, Faculty of Medicine, Graduate School of Medicine, Hokkaido University, N-15, W-7, Kita-ku, Sapporo, Hokkaido 060-8638 Japan; 2 Sapporo Diabetes and Thyroid Clinic, Sapporo, Hokkaido Japan; 3 Division of Diabetes and Endocrinology, Department of Medicine, NTT Sapporo Medical Center, Sapporo, Hokkaido Japan; 4 Caress Sapporo Hokko Memorial Clinic, Sapporo, Hokkaido Japan

**Keywords:** Pemafibrate, Liver function, Type 2 Diabetes

## Abstract

**Background:**

Pemafibrate has been reported to ameliorate lipid profiles and liver dysfunction. However, which patients derive benefit from the hepatoprotective effects of pemafibrate is unclear.

**Methods:**

We conducted a sub-analysis of the PARM-T2D study where subjects with type 2 diabetes complicated by hypertriglyceridemia were prospectively treated with pemafibrate or conventional therapies for 52 weeks. From the original cohort, subjects who had metabolic-associated fatty liver disease without changing their treatment regimens for comorbidities were analyzed. Eligible subjects (n = 293) (average age 61.2 ± 11.7 years, 37.5% female) treated with pemafibrate (pemafibrate, n = 152) or controls who did not change their treatment regimens (controls, n = 141) were divided into three groups based on their alanine aminotransferase (ALT) levels: ALT ≤ upper normal limit (UNL) (pemafibrate, n = 65; controls, n = 50), UNL < ALT ≤ 2×UNL (pemafibrate, n = 58; controls, n = 54), and 2×UNL < ALT (pemafibrate, n = 29; controls, n = 27).

**Results:**

Pemafibrate treatment significantly ameliorated ALT levels (from 29 to 22 U/L, *p* < 0.001 by Wilcoxon’s signed-rank test) in the total cohort and subjects with high ALT levels (2×ULN < ALT), and improved liver fibrosis as assessed by the Fibrosis-4 index (mean change − 0.05 (95% confidence interval: −0.22 to − 0.02), *p* < 0.05 versus baseline by the Mann-Whitney *U*-test and *p* < 0.05 versus the ALT ≤ UNL group by the Kruskal–Wallis test followed by Dunn’s post-hoc analysis).

**Conclusions:**

The hepatoprotective effects of pemafibrate were dominant in subjects with type 2 diabetes complicated with liver dysfunction.

**Trial registration:**

This study was registered with the University Hospital Medical Information Network Center Clinical Trials Registry (UMIN000037385).

**Supplementary Information:**

The online version contains supplementary material available at 10.1186/s13098-023-01187-7.

## Background

Managing metabolic complications in subjects with type 2 diabetes (T2D) is a key issue for achieving a better quality of life and life expectancy [1]. Among diabetic complications, metabolic-associated fatty liver disease (MAFLD) is an important liver comorbidity and the principal cause of liver disease worldwide [2]. Conversely, subjects with progressive liver fibrosis have a higher incidence of T2D [3], indicative of a close relationship between T2D and MAFLD. In addition, T2D was identified as a significant risk factor among several metabolic abnormalities for the development of non-alcoholic steatohepatitis (NASH) and the progression of liver fibrosis in women with NASH [4]. T2D and MAFLD/NASH are associated with vascular risk and cardiovascular disease progression, and insulin resistance mainly caused by obesity and metabolic disorders is the underlying common mechanism between these diseases [5]. Several metabolic disorders that accompany diabetes can lead to hepatocellular damage; therefore, treatment strategies with hepatoprotective effects are required.

Pemafibrate, a selective peroxisome proliferator-activated receptor α modulator, was shown to exert hepatoprotective effects in previous phase II/III studies comprising subjects with hypertriglyceridemia [6, 7]. However, real-world evidence in subjects already treated with fibrates, with a focus on subjects with T2D, is needed. In the present study, we evaluated the effects of pemafibrate on MAFLD complicated with T2D in a real-world clinical setting.

## Methods

### Study design and participants

This was a secondary analysis of data derived from a multi-center prospective observational study (the PARM-T2D study) [8]. Briefly, 685 adults with T2D and hypertriglyceridemia, including individuals who were or were not taking a conventional fibrate, were enrolled. After providing written informed consent, the participants were treated with pemafibrate 0.2–0.4 mg/day or continued their existing treatment for hyperlipidemia. Blood and urine samples were collected after overnight fasting and physical assessments were performed at baseline, and then repeated 52 weeks after the start of the study.

For the present sub-analysis, participants who changed their treatments for comorbidities were excluded to minimize the confounding effects on MAFLD. In addition, subjects who were unlikely to have fatty liver (fatty liver index (FLI) < 30) and lacked relevant values were excluded. The remaining subjects were divided into three groups based on their baseline alanine aminotransaminase (ALT) levels (ALT ≤ upper limit of normal (ULN), ULN < ALT ≤ 2×ULN, and 2×ULN < ALT) and changes in liver enzymes were also compared among the groups. Effects on liver fibrosis were assessed using the Fibrosis-4 (FIB-4) index. The FLI and FIB-4 index were calculated as described elsewhere [9] [10].

The PARM-T2D study was registered with the University Hospital Medical Information Network Center Clinical Trials Registry (UMIN000037385) and approved by the institutional review board of Hokkaido University Hospital (018–0440). The study was performed in accordance with the principles of the Declaration of Helsinki and its amendments.

### Statistical analysis

Normally distributed continuous data are expressed as the mean ± SD, non-normally distributed continuous data are expressed as the median (quartiles), and categorical data are expressed as a number (%). Comparisons of two groups were made using the unpaired *t*-test or Mann-Whitney *U*-test for continuous variables, and the chi-square test for categorical variables. Results within groups were compared by a paired *t*-test or Wilcoxon’s signed-rank test. Changes in variables from baseline are expressed as the mean or median (95% confidence interval [CI]), and the groups were compared using the Kruskal–Wallis test, followed by Dunn’s post-hoc analysis. Relationships between variables were evaluated using Spearman’s rank correlation analysis. *P* < 0.05 indicated statistical significance. Data were analyzed using GraphPad Prism 8.4.2 (GraphPad Software, Inc. San Diego, CA, USA).

## Results

**Fig. 1:**
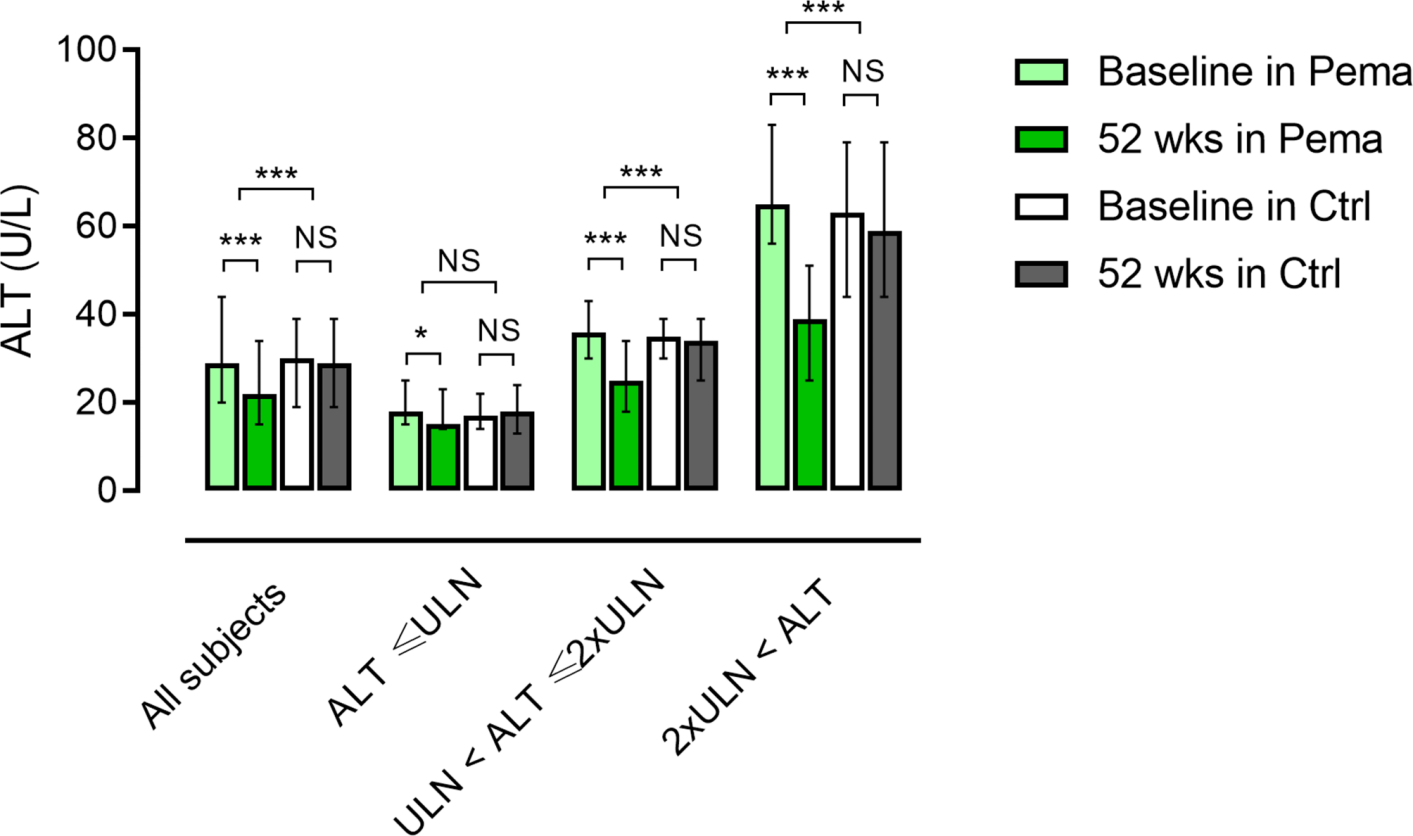
Changes in alanine transaminase before and at the end of the study period Bars are the median (25–75%). * *p* < 0.05, *** *p* < 0.001 between 0 and 52 weeks (Wilcoxon’s signed-rank test for within-group comparisons, and Mann-Whitney *U*-test for changes between groups). Light and dark green bars represent 0 and 52 weeks in the pemafibrate group, and white and gray bars represent 0 and 52 weeks in the control group. ALT, alanine aminotransaminase; ULN, upper limit of normal, Pema, pemafibrate, Ctrl, control, NS, not significant

Overall, 548 out of 685 participants completed the original study. After excluding 164 subjects who changed treatment regimens for comorbidities, 93 subjects were additionally exempt from this analysis because of low FLI and/or a lack of relevant values. As a result, the remaining participants comprising the pemafibrate group (n = 152) and control group (n = 141) were analyzed (Supplementary Fig. 1). The mean age of all participants was 61.2 ± 11.7 years and 37.5% were female. There were no significant differences in baseline characteristics between the two groups (Table 1). After 52 weeks, the ALT level was significantly improved in the pemafibrate group (from 29 to 22 U/L, *p* < 0.001) in the total cohort but not in the control group (Fig. 1), similar to other deviated liver enzymes (Supplementary Fig. 2). Thereafter, these participants were divided into three groups: ALT ≤ ULN (pemafibrate n = 65, control n = 60), ULN < ALT ≤ 2×ULN (pemafibrate n = 58, control n = 54), and 2×ULN < ALT (pemafibrate n = 29, control n = 27) (Table 1). Notably, such efficacy was more distinct in subjects with higher liver enzyme elevation at baseline (Fig. 1 and Supplementary Fig. 2). Regarding liver fibrosis, the FIB-4 index significantly deteriorated in the control group (from 1.32 to 1.37, *p* = 0.036), whereas there was no change in the pemafibrate group (Supplementary Fig. 3). The analysis of the ALT subgroup indicated no significant changes in the FIB-4 index in each subgroup of the control group. However, pemafibrate ameliorated the FIB-4 index in subjects with severe liver dysfunction (2×ULN < ALT) (Table 2), but this improvement was not statistically significant when compared with the control group (Supplementary Fig. 4). To investigate which clinical parameters correlated with an improvement in liver fibrosis, we performed correlation analysis using changes in the FIB-4 index and other parameters. As shown in Table 3, changes in low-density lipoprotein cholesterol (LDL-C) and LDL-C/apolipoprotein B (apoB) were positively correlated with an improvement in the FIB-4 index. However, changes in other metabolic parameters did not correlate with changes in the FIB-4 index.

**Table 1:** Demographic and clinical characteristics of the participants at baseline

Variables	Pemafibrate (n = 152)	Control (n = 141)	*P*-value
Age	60.9 ± 11.8	61.4 ± 11.5	0.723
Female sex (n, (%))	53 (34.9)	57 (40.4)	0.326
Duration of diabetes (n, (%))			0.430
<5 years	31 (20.4)	34 (24.1)	
>5–15 years	64 (42.1)	64 (45.4)	
>15 years	57 (37.5)	43 (30.5)	
Body mass index (kg/m^2^)	27.9 ± 3.9	28.1 ± 4.3	0.777
HbA1c (%)	7.04 ± 0.78	7.03 ± 0.81	0.952
AST (U/L)	31.1 ± 17.3	29.0 ± 14.8	0.260
ALT (U/L)	29 (20, 44)	30 (19, 39)	0.531
ALT ≤ ULN (U/L)	18 (15, 25)	17 (14, 22)	0.408
ULN < ALT ≤ 2× ULN (U/L)	36 (30, 43)	35 (30, 39)	0.389
2× ULN < ALT (U/L)	65 (56, 83)	63 (44, 79)	0.372
γ-GTP (U/L)	41 (25, 72)	44 (30, 60)	0.776
eGFR (mL/min/1.73m^2^)	69.3 ± 18.9	69.4 ± 23.0	0.963
Triglyceride (mg/dL)	186 (145, 214)	185 (155, 230)	0.819
HDL-C (mg/dL)	51.5 ± 12.8	50.7 ± 11.9	0.588
LDL-C (mg/dL)	100.9 ± 25.7	105.3 ± 31.0	0.191
Hypertension (n, (%))	98 (64.5)	91 (64.5)	0.991
Cardiovascular diseases (n, (%))	13 (8.6)	9 (6.4)	0.481
Fibrate use (n, (%))	53 (34.9)	37 (26.2)	0.110

Values are the mean ± SD, median (25, 75%), or number (%). *P*-values for the Pemafibrate vs. Control groups were obtained by Student’s *t*-test, Mann Whitney *U-*test, or chi-square test. HbA1c, glycated hemoglobin; AST, aspartate aminotransaminase; ALT, alanine aminotransaminase; γ-GTP, γ-glutamyl transpeptidase, eGFR, estimated glomerular filtration rate, HDL-C, high-density lipoprotein cholesterol, LDL-C, low-density lipoprotein cholesterol

**Table 2:** Changes in the FIB-4 index during the study period

		ALT ≦ ULN	ULN < ALT ≦ 2×ULN	2×ULN < ALT	*P*-value for changes among the groups
**Pemafibrate**	**Baseline**	1.18 (0.92, 1.61)	1.42 (0.95, 1.85)	1.76 (1.10, 2.01)	
	**Changes**	0.02 (− 0.04 to 0.09)	0.07 (− 0.09 to 0.12)	−0.05 (− 0.22 to − 0.02) *†	0.036
**Control**	**Baseline**	1.41 (0.97, 1.92)	1.23 (0.95, 1.70)	1.26 (0.70, 1.94)	
	**Changes**	0.04 (− 0.03 to 0.10)	0.06 (− 0.01 to 0.10)	0.02 (− 0.19 to 0.20)	0.884

Values are the median (25, 75%) or median change (95% CI). *p < 0.05 vs. baseline (Mann-Whitney *U*-test), †*p* < 0.05 vs. ALT ≤ ULN group (Dunn’s test). ALT, alanine aminotransaminase; ULN, upper limit normal

**Table 3:** Relationships between changes in the Fib-4 index in the pemafibrate group and changes in metabolic parameters over the 52-week study period

	ΔFib-4 index	
Variables	ρ	*P* value
ΔBMI	0.014	0.865
ΔTriglyceride	0.098	0.229
ΔHDL-C	0.018	0.817
ΔLDL-C	−0.185	0.023
ΔRLP-C	0.079	0.347
ΔApoA1	0.051	0.548
ΔApoB	−0.075	0.377
ΔApoE	0.113	0.179
ΔLDL-C/apoB	−0.204	0.015
Δγ-GTP	0.060	0.459
ΔeGFR	−0.019	0.815
ΔHbA1c	−0.063	0.443

*P*-values were obtained by Spearman’s rank correlation analysis. BMI, body mass index; HDL-C, high-density lipoprotein cholesterol; LDL-C, low-density lipoprotein cholesterol; RLP-C, remnant-like particle cholesterol; ApoA1, apolipoprotein A1; ApoB, apolipoprotein B; ApoE, apolipoprotein E; γ-GTP, γ-glutamyl transpeptidase; eGFR, estimated glomerular filtration rate; Hb1c, glycated hemoglobin

## Discussion

In the present study, we found that pemafibrate treatment significantly ameliorated liver dysfunction and that this efficacy was distinct in subjects with a higher liver enzyme elevation. In addition, liver fibrosis appeared to have been improved in this population. Pemafibrate specifically activates target genes related to lipid metabolism in the liver, thus avoiding the activation of undesirable genes caused by off-target effects as observed for other fibrates [11]. Furthermore, the upregulation of fibroblast growth factor-21 (FGF-21), a hormone primarily expressed by the liver and adipose tissue, was closely related to hepatic metabolic pathways [12]. Pemafibrate treatment increased serum FGF-21 levels ^[7]^, leading to improved liver function [8, 13] and the alleviation of inflammation and steatosis of the liver [14, 15].

A notable finding of our study was that pemafibrate improved the FIB-4 index, a factor that reflects liver fibrosis, which correlated with changes in the LDL profiles. A previous phase II trial of subjects with MAFLD revealed that 72 weeks of pemafibrate treatment did not decrease the liver fat content but significantly reduced liver stiffness [16]. The administration of pemafibrate to NASH diet-fed mice also showed an improvement in liver fibrosis *via* a reduction of intrahepatic cholesterol but not intrahepatic triglyceride [17]. LDL-C/apoB conventionally represents an alternative index of LDL particle size. As shown in real-world trials [8, 18], pemafibrate increased serum LDL-C levels without changing the apoB level, indicating an infrequency of toxic small dense LDL (sd-LDL) particles in the plasma. Although a precise interactional mechanism has not been determined, considering the close relationship between serum sd-LDL and liver fibrosis [19, 20], the amelioration of LDL function by pemafibrate might have benefit for patients with liver fibrosis.

The limitations of the original trial have been described previously [8]. Additionally, this subanalysis had a smaller number of participants than the original study because it focused on subjects with MAFLD, which might have made the efficacy of pemafibrate on liver dysfunction clearer. We used the FLI and FIB-4 index as markers of liver steatosis and fibrosis, respectively. Unfortunately, pathological examination, the standard method for measuring liver fibrosis, was not available. A further randomized, controlled trial that includes the assessment of changes in liver pathology with a focus on subjects with T2D would therefore be needed.

## Conclusions

Pemafibrate exerted a hepatoprotective action by changing the serum lipid profiles of subjects with MAFLD complicated with T2D, especially those with higher liver enzyme elevation.

### Electronic supplementary material

Below is the link to the electronic supplementary material.

Supplementary Material 1

Supplementary Material 2

Supplementary Material 3

Supplementary Material 4

## Data Availability

Analysis data are available from the corresponding author upon reasonable request.
