# Supplementary figures and images for: Preferable effects of pemafibrate on liver function and fibrosis in subjects with type 2 diabetes complicated with liver damage
Source: Diabetol Metab Syndr. 2023 Oct 26;15:214. doi: 10.1186/s13098-023-01187-7 (PMC10601300; doi:10.1186/s13098-023-01187-7)

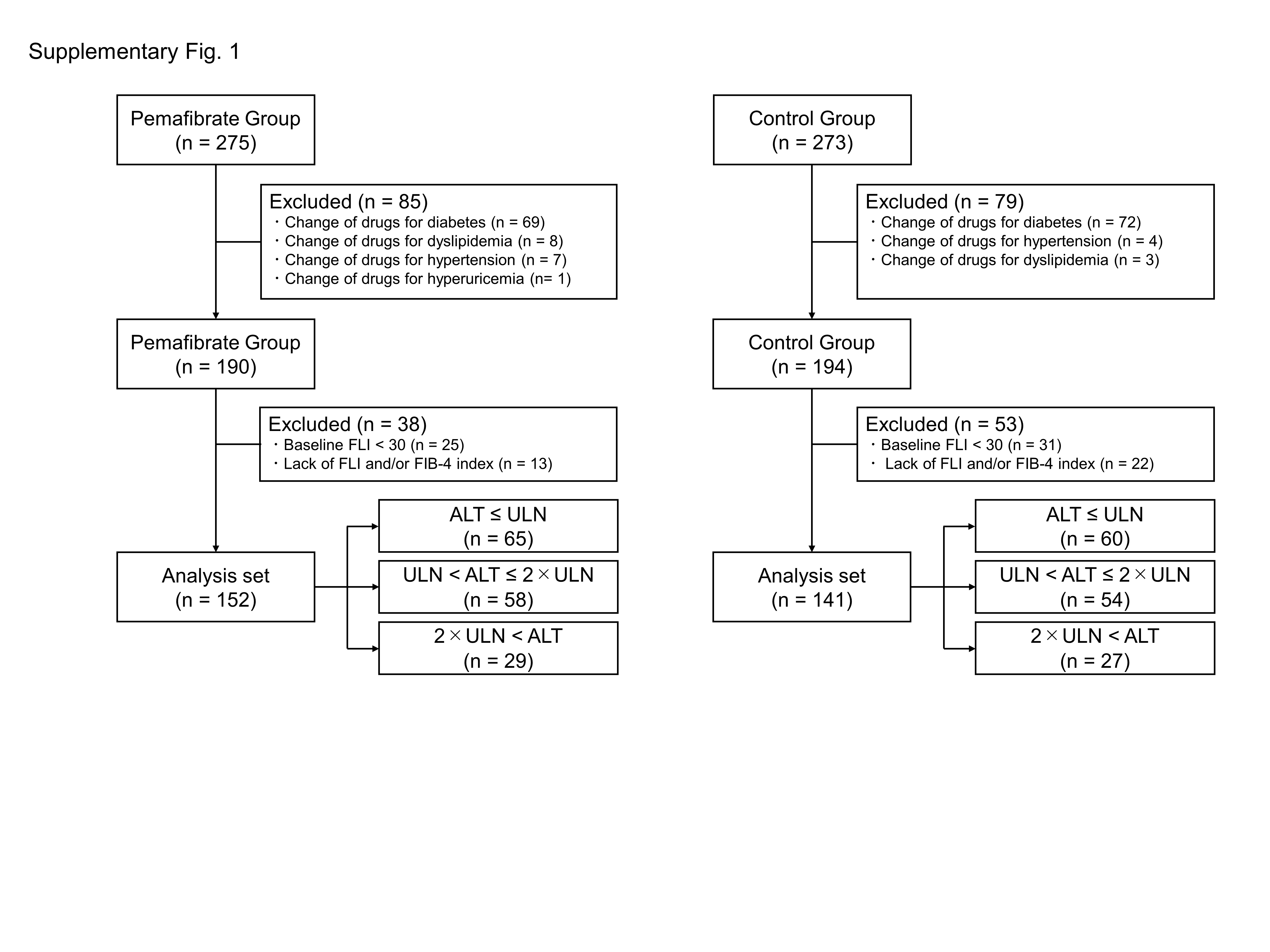

Supplement: Supplementary file 1 — Supplementary Material 1 [file 13098_2023_1187_MOESM1_ESM.tif]

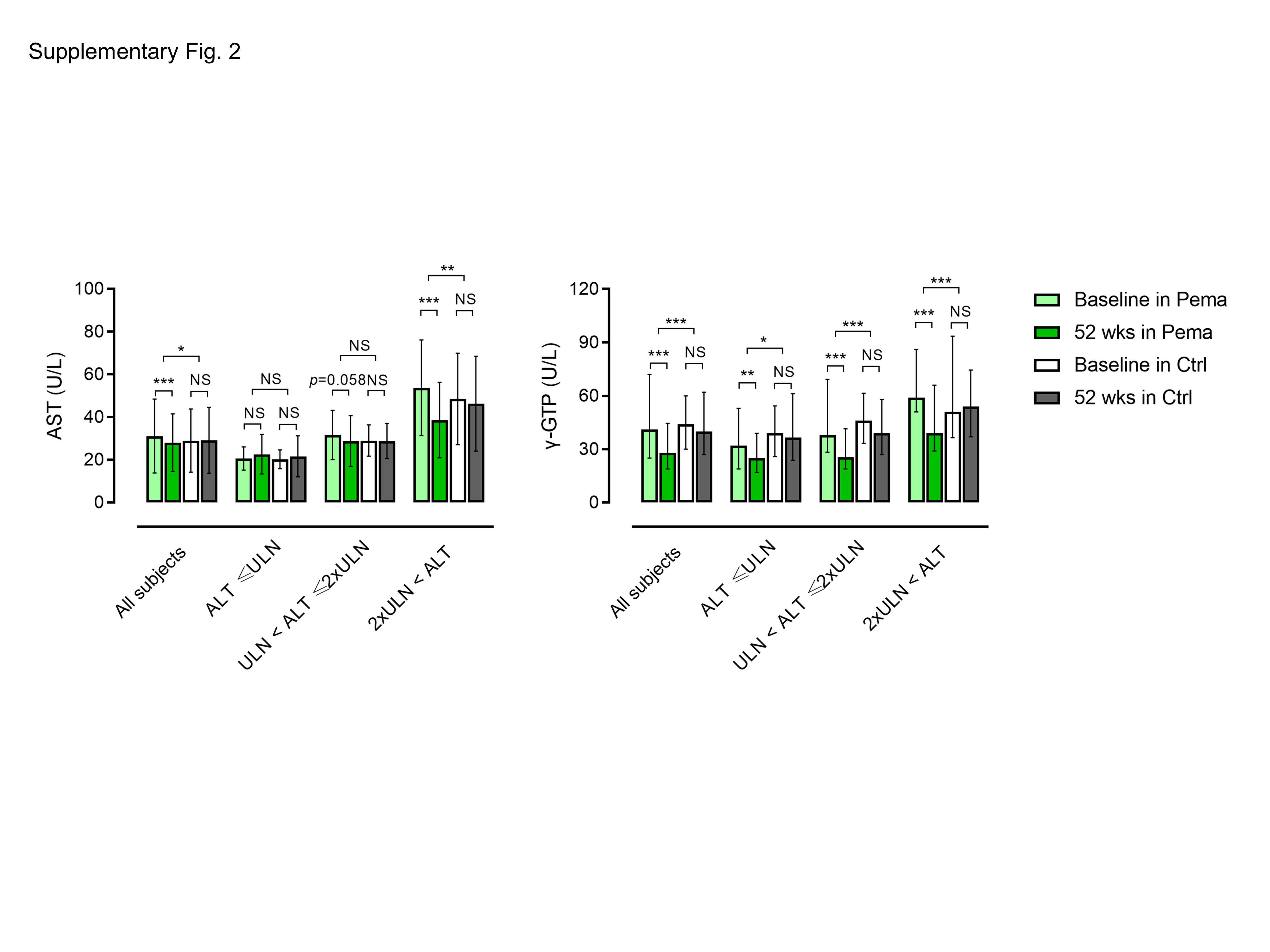

Supplement: Supplementary file 2 — Supplementary Material 2 [file 13098_2023_1187_MOESM2_ESM.tif]

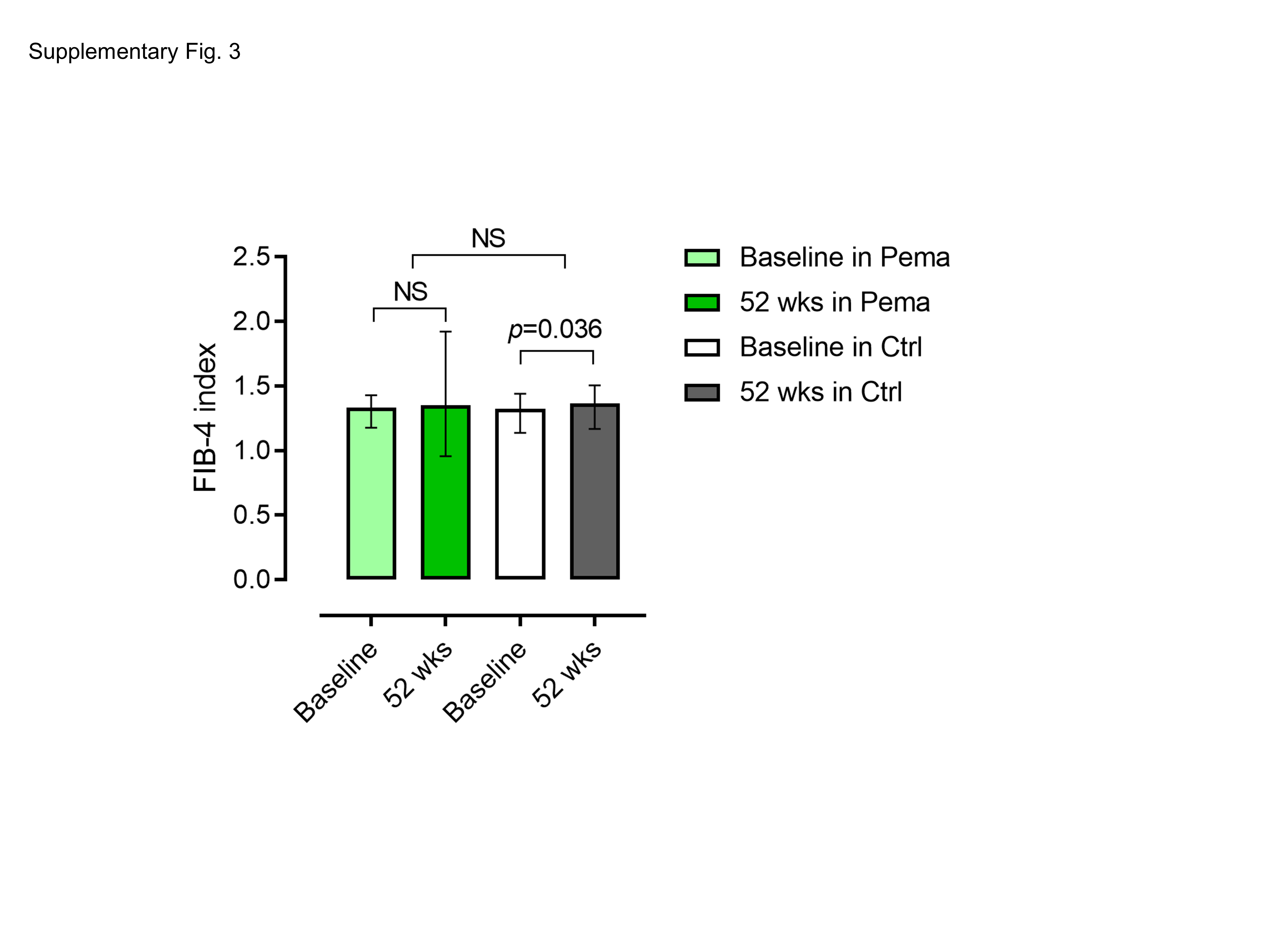

Supplement: Supplementary file 3 — Supplementary Material 3 [file 13098_2023_1187_MOESM3_ESM.tif]

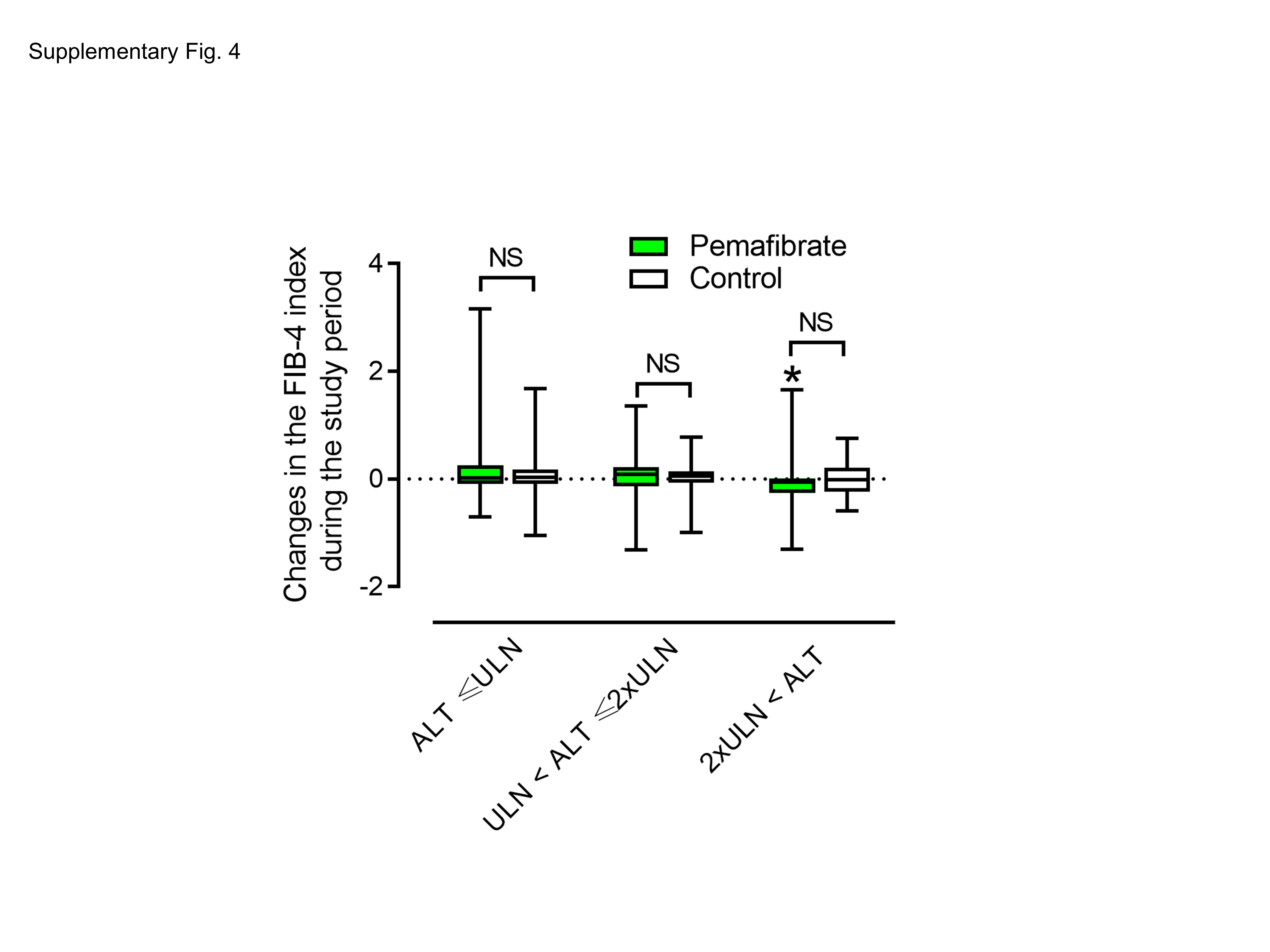

Supplement: Supplementary file 4 — Supplementary Material 4 [file 13098_2023_1187_MOESM4_ESM.tif]
